# Amplification and sequencing of entire tick mitochondrial genomes for a phylogenomic analysis

**DOI:** 10.1038/s41598-022-23393-5

**Published:** 2022-11-11

**Authors:** Alexander R. Kneubehl, Sebastián Muñoz-Leal, Serhii Filatov, Daniel G. de Klerk, Ronel Pienaar, Kimberly H. Lohmeyer, Sergio E. Bermúdez, Thanchira Suriyamongkol, Ivana Mali, Esther Kanduma, Abdalla A. Latif, M’hammed Sarih, Ali Bouattour, Adalberto A. Pérez de León, Pete D. Teel, Marcelo B. Labruna, Ben J. Mans, Job E. Lopez

**Affiliations:** 1 Department of Pediatrics, National School of Tropical Medicine, Baylor College of Medicine, Houston, TX USA; 2 Departamento de Ciencia Animal, Facultad de Ciencias Veterinarias, Universidad de Concepción, Chillán, Chile; 3 Agricultural Research Council-Onderstepoort Veterinary Research, Pretoria, South Africa; 4 Knipling-Bushland U.S. Livestock Insects Research Laboratory, United States Department of Agriculture-Agricultural Research Service, Kerrville, TX USA; 5 Medical Entomology Department, Gorgas Memorial Institute for Health Research, City of Panamá, Panama; 6 Department of Biology, Eastern New Mexico University, Portales, NM USA; 7 Present Address: Southern Illinois University-Carbondale, Cooperative Wildlife Research Laboratory, Carbondale, IL USA; 8 Fisheries, Wildlife, and Conservation Biology Program, North Carolina State University, Raleigh, USA; 9 Department of Biochemistry, Faculty of Science and Technology, University of Nairobi, Nairobi, Kenya; 10 University of KwaZulu-Natal, School of Life Sciences, Durban, Westville South Africa; 11 Institut Pasteur du Maroc, Service de Parasitologie et des Maladies Vectorielles, Casablanca, Morocco; 12 Institut Pasteur de Tunis, Université Tunis El Manar, Laboratoire Virus, Vecteurs, Hôtes, Service d’Entomologie Médicale, Tunis, Tunisia; 13 San Joaquin Valley Agricultural Sciences Center, United States Department of Agriculture-Agricultural Research Service, Parlier, CA USA; 14 Department of Entomology, Texas A&M AgriLife Research, College Station, TX USA; 15 Departamento de Medicina Veterinária Preventiva e Saúde Animal, Faculdade de Medicina Veterinária e Zootecnia, Universidade de São Paulo, São Paulo, Brazil; 16 Department of Life and Consumer Sciences, University of South Africa, Pretoria, South Africa; 17 Department of Veterinary Tropical Diseases, University of Pretoria, Pretoria, South Africa; 18 Department of Molecular Virology and Microbiology, Baylor College of Medicine, Houston, TX USA

**Keywords:** Comparative genomics, Population genetics, DNA sequencing, Bioinformatics, Eukaryote, Genetics, Comparative genomics, Entomology, Phylogenetics, Population genetics, Taxonomy

## Abstract

The mitochondrial genome (mitogenome) has proven to be important for the taxonomy, systematics, and population genetics of ticks. However, current methods to generate mitogenomes can be cost-prohibitive at scale. To address this issue, we developed a cost-effective approach to amplify and sequence the whole mitogenome of individual tick specimens. Using two different primer sites, this approach generated two full-length mitogenome amplicons that were sequenced using the Oxford Nanopore Technologies’ Mk1B sequencer. We used this approach to generate 85 individual tick mitogenomes from samples comprised of the three tick families, 11 genera, and 57 species. Twenty-six of these species did not have a complete mitogenome available on GenBank prior to this work. We benchmarked the accuracy of this approach using a subset of samples that had been previously sequenced by low-coverage Illumina genome skimming. We found our assemblies were comparable or exceeded the Illumina method, achieving a median sequence concordance of 99.98%. We further analyzed our mitogenome dataset in a mitophylogenomic analysis in the context of all three tick families. We were able to sequence 72 samples in one run and achieved a cost/sample of ~ $10 USD. This cost-effective strategy is applicable for sample identification, taxonomy, systematics, and population genetics for not only ticks but likely other metazoans; thus, making mitogenome sequencing equitable for the wider scientific community.

## Introduction

Ticks (Acari: Ixodida) comprise over 900 species of obligate hematophagous arthropods many of which are important vectors of pathogens for both humans and livestock^1,2^. Furthermore, tick bites are capable of eliciting a variety of acute and long-term conditions, not caused by an infectious agent, such as tick toxicosis^3–5^ and alpha-gal syndrome (red meat allergy)^6^. Given the health concerns associated with these arthropods, surveillance and investigations into mechanisms of dispersal are necessary. One approach to investigate tick dispersal is through analysis of mitochondrial genomes (mitogenomes).

The mitogenome has great utility for molecular analyses as it is haploid, does not undergo recombination, and exists in greater abundance relative to the nuclear genome^7^. The abundance of the mitogenome allows for easier amplification or direct sequencing from degraded samples^8,9^. Advances in mitogenome sequencing of individual tick specimens pushed tick taxonomy and systematics to new heights in the last few years^10–13^, and have expanded our ability to perform population genetics studies to investigate tick dispersal and distribution^13,14^. Current strategies to sequence tick mitogenomes largely revolve around genome skimming, which is low-coverage sequencing of a genomic DNA sample and recovering reads to completely assemble both the mitogenome and nuclear loci^10,11,13–16^. An additional strategy is amplification and sequencing of the mitogenome in multiple amplicons^12,17–19^. While successful, these approaches are expensive and impractical in resource limited settings, for large scale studies (e.g. population genetics), and for low throughput studies where the cost per sample is less advantageous for next-generation sequencing. To this end, we sought to develop a workflow that would be as accurate as Illumina sequencing but comparable in price to single-amplicon Sanger sequencing.

We present here a direct strategy to amplify, sequence, and annotate the mitogenome from individual tick samples utilizing Oxford Nanopore Technologies’ (ONT) MinION Mk1B platform. To demonstrate the strength of this strategy we successfully sequenced the complete or nearly complete mitogenomes of 85 individual tick samples. Forty-three of these samples had no prior complete mitogenome sequence on GenBank for the given species. We benchmarked our approach using samples previously sequenced with an Illumina genome skimming strategy^10^. The utility of this new strategy was demonstrated in a mitophylogenomic analysis of all three tick families (Ixodidae, Argasidae, and Nuttalliellidae). This strategy will be important for use in generating the necessary mitogenome datasets for population genetics studies, taxonomic and systematics investigations, and for tick surveillance efforts. Moreover, this strategy likely has applications for other metazoan organisms.

## Results

### Primer design and full-length amplification of tick mitogenomes

**Figure 1:**
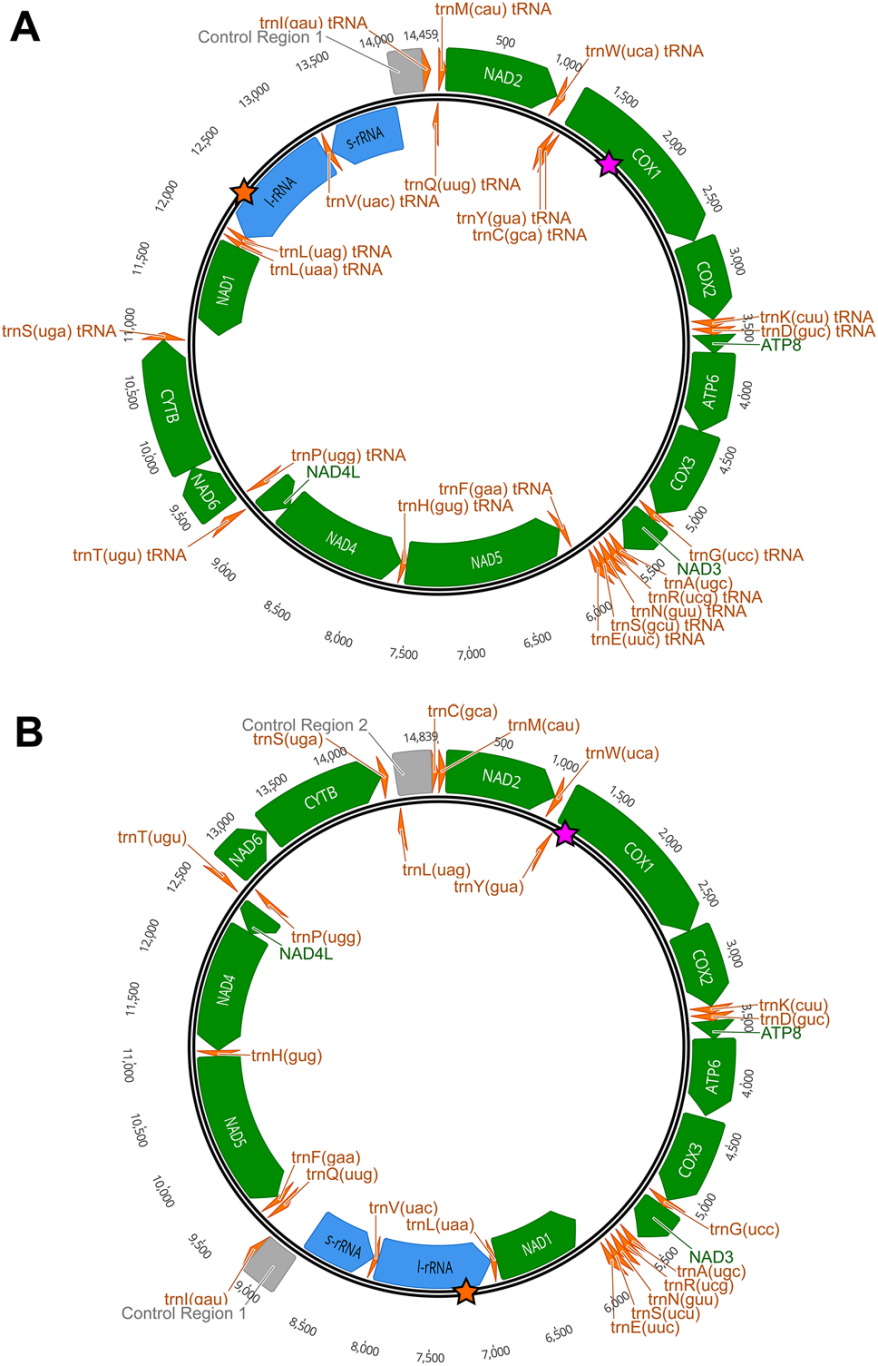
Mitogenome organizations of Group A and B ticks. Representative mitogenome organizations are depicted for what we termed Group A tick taxa, Argasidae, Nuttalliellidae, Prostriates (**A**), and Group B taxa, Metastriates (**B**). Protein coding genes are colored green and are labeled with their gene acronym. Transfer RNAs are colored orange while ribosomal RNAs are colored blue. The non-coding controls regions are indicated with grey boxes. Gene feature directions are indicated by location either above (positive-sense) or below (negative-sense) the mid-line. Primer set 1 was designed in the *cox1* gene (pink star) and primer set 2 was designed in the 16S rRNA gene (orange star) of both groups.

The genetic organization of Group A (argasids, nuttallielids, and Prostriates) and Group B (Metastriates) mitogenomes differ (Fig. 1)^15,20–22^. Consequently, two different degenerate primer sets (Supplementary Table S1) were designed for each tick group. We refer to these generically as primer set 1 (P1) and primer set 2 (P2) throughout this work. Each primer set generated a full-length mitogenome amplicon of ~ 15 kb. We tested these primers on 163 specimens from all three tick families with 16 genera, and 79 species represented (Supplementary Table S2). The samples tested included multiple representatives per species and samples from different localities, when available. Of the 163 samples, 115 samples amplified with at least one primer set and 73 of these samples amplified with both primer sets. At the species-level, we amplified the mitogenome with at least one primer set for 67 species and 50 of those species with both primer sets.

We also developed species-specific primers (Supplementary Table S1). This was needed for species that already had a mitogenome available on GenBank but did not amplify with either primer set (e.g. *Chiropterargas boueti*) and for species that did not have an available mitogenome but did amplify with only one primer set (e.g. *Ixodes angustus*). For species with an available mitogenome, we took P1 and P2 primer set sequences and performed a BLASTn analysis against the available sequence data of the given species on GenBank. Modifications were made when necessary to decrease mismatches in primer sequences. For species that amplified with a single primer set, these were sequenced (see “Sequencing and assembly”) and a draft assembly was created. We aligned the primer set that did not initially amplify or where the PCR yield was low to the draft assembly. Primer sequences were then modified to reflect the sequence in the draft assemblies. The species-specific primers (ssP1 and ssP2) (Supplementary Table S1) were used with the samples indicated in Supplementary Table S3. The species-specific primers were successful in amplifying 12 of 30 samples’ ssP1 amplicons and four of 11 samples’ ssP2 amplicons. Species-level success for amplicons that were generated by both primer sets was increased from 50 to 62.

### Sequencing and assembly

We prioritized 87 samples for ONT sequencing. This was based on the following criteria: (1) a sample that amplified robustly with one or both primer sets; (2) a sample that was previously sequenced by Mans et al*.*^10^; and (3) species that did not have a mitogenome sequence in GenBank. If a complete mitogenome was not available on GenBank, two specimens were sequenced when possible. Sequencing was performed using the SQK-RBK004 (6 samples) or SQK-RBK110.96 (81 samples) library prep kits. Two different SQK-RBK110.96 libraries were performed. The first library SQK-RBK110.96 (SQK-RBK110.96-1) included samples that only amplified with one primer set and were used as draft assemblies to design species-specific primers. The SQK-RBK110.96-2 library sequenced the amplicons from the species-specific primers and included more tick specimens. Collectively, the sequencing libraries yielded average read lengths between 1.5 kb and 4.1 kb (Supplementary Table S4). On average 2,186 reads and 6.84 Mb were produced per sample. Of the 87 samples sequenced, 85 produced a final assembly that was considered for analysis (Supplementary Table S4). The criterion for analysis was based on whether the assembly product was > 14 kb and < 16 kb. The final assemblies had a range in mean depth of coverage from 96× to 1460×.

### Mitogenome annotation

Assemblies were annotated using a combined automated and manual approach. The final assemblies were first annotated using MitoZ^23^, and then manually edited to reduce gene overlap and address frameshift errors. The MitoZ software commonly annotated overlaps in the *nad1* gene and the adjacent *trnS* gene for Group A organisms. There was one case where MitoZ failed to annotate a protein coding gene despite the presence of a complete open reading frame in the appropriate location (*atp8* in the *Haemaphysalis longicornis* sample), and manual annotation corrected this error. Manual annotation also included using ARWEN^24^, which identified and predicted tRNA genes that MitoZ missed. Furthermore, manual annotation corrected homopolymer errors that disrupted protein coding genes (i.e. frameshift errors causing premature stop codons). Homopolymer errors were almost exclusively the reason for premature stop codons in the protein coding genes. During manual annotation, we observed that those samples that amplified with only one primer set (Supplementary Table S2) contained either a compromised *cox1* gene (primer site 1) or 16S gene (primer site 2) for P1- or P2-only amplification, respectively.

### Benchmarking ONT assemblies against Illumina reference

**Figure 2:**
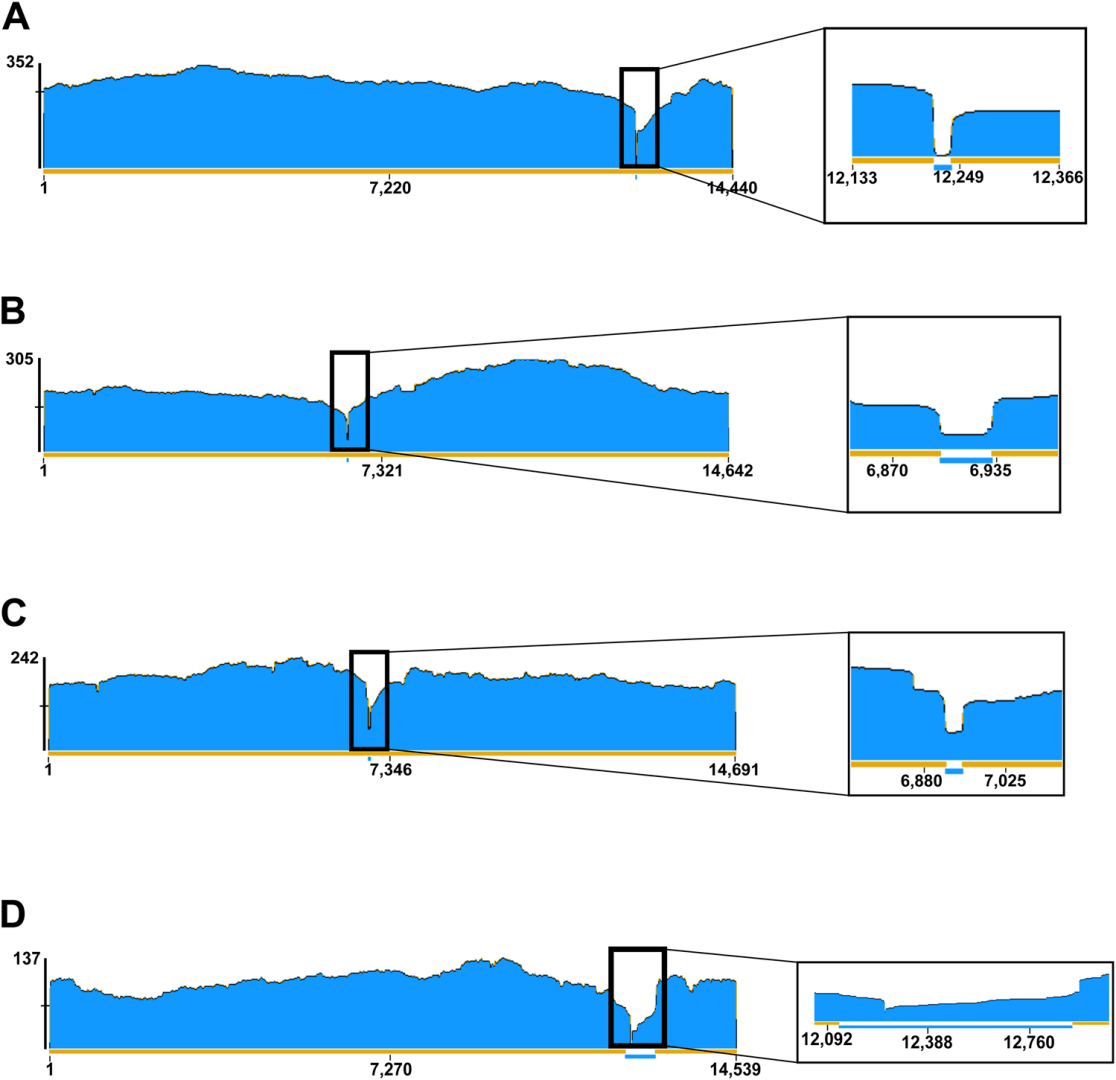
Sequence coverage analysis. To investigate if read coverage was the reason for the increased amount of nucleotide differences observed between our ONT assemblies and the Illumina references, we mapped our ONT reads against the Illumina assemblies. The read-depth coverage histogram generated by Geneious Prime is depicted for each sample of concern*, Argas africolumbae* JM2 (**A**), *Amblyomma tholloni* 1 (**B**), *Rhipicephalus zambeziensis* (**C**), and *Ixodes rubicundus* (**D**). The inset panels show a zoomed in look at the lower coverage areas. Nucleotide positions across the mitogenome are indicated. The max read-depth coverage is indicated to the left of the histogram. The gold and blue bars at the bottom of the coverage histogram indicate positions above ×60 and below ×60 read coverage, respectively.

To determine the accuracy of ONT-generated mitogenome assemblies, we performed a benchmarking analysis. From the 87 samples that were sequenced, Mans et al.^10^ had previously sequenced 15 using an Illumina genome skimming approach. We compared the ONT assemblies of these samples (prior to manual frameshift-error correction) to the Illumina assembly from GenBank using NucDiff^25^. The uncorrected ONT assemblies had a 99.80% and 99.93% mean and median sequence accuracy, respectively (Supplementary Table S5). Two assemblies that amplified with only one primer set had most nucleotide differences at the ends of the amplicon. We masked the areas on the flanks of the partial mitogenome that had poor read coverage (< 60x). The number of nucleotide differences was greatly reduced after masking, indicating most nucleotide differences were introduced at the amplicon ends that had poor read coverage (Fig. 2). By read mapping to the Illumina reference, we confirmed that the area surrounding the P2 site was poorly represented in the sequencing data for *Argas africolumbae* JM2 (Fig. 2A). When we masked this region in *A. africolumbae* JM2 the sequence concordance with the Illumina reference rose from 99.72 to 99.97%. (Supplementary Table S5). However, there were assemblies that had a high number of nucleotide differences even when both amplicons were sequenced (e.g. *Amblyomma tholloni* 1, *Ixodes rubicundus*, *Rhipicephalus zambeziensis*). We confirmed that the *A. tholloni* 1 (Fig. 2B) and *R. zambeziensis* (Fig. 2C) samples had a read depth of coverage throughout most of the assembly that was greater than 100×. However, only one amplicon for the *I. rubicundus* sample sequenced well, which coupled with the low coverage for that amplicon created a 590 bp region of poor read depth of coverage (< 60×) surrounding the P2 site (Fig. 2D). Indeed, approximately 60% of nucleotide differences in the ONT *I. rubicundus* assembly occurred in this area.

**Figure 3:**
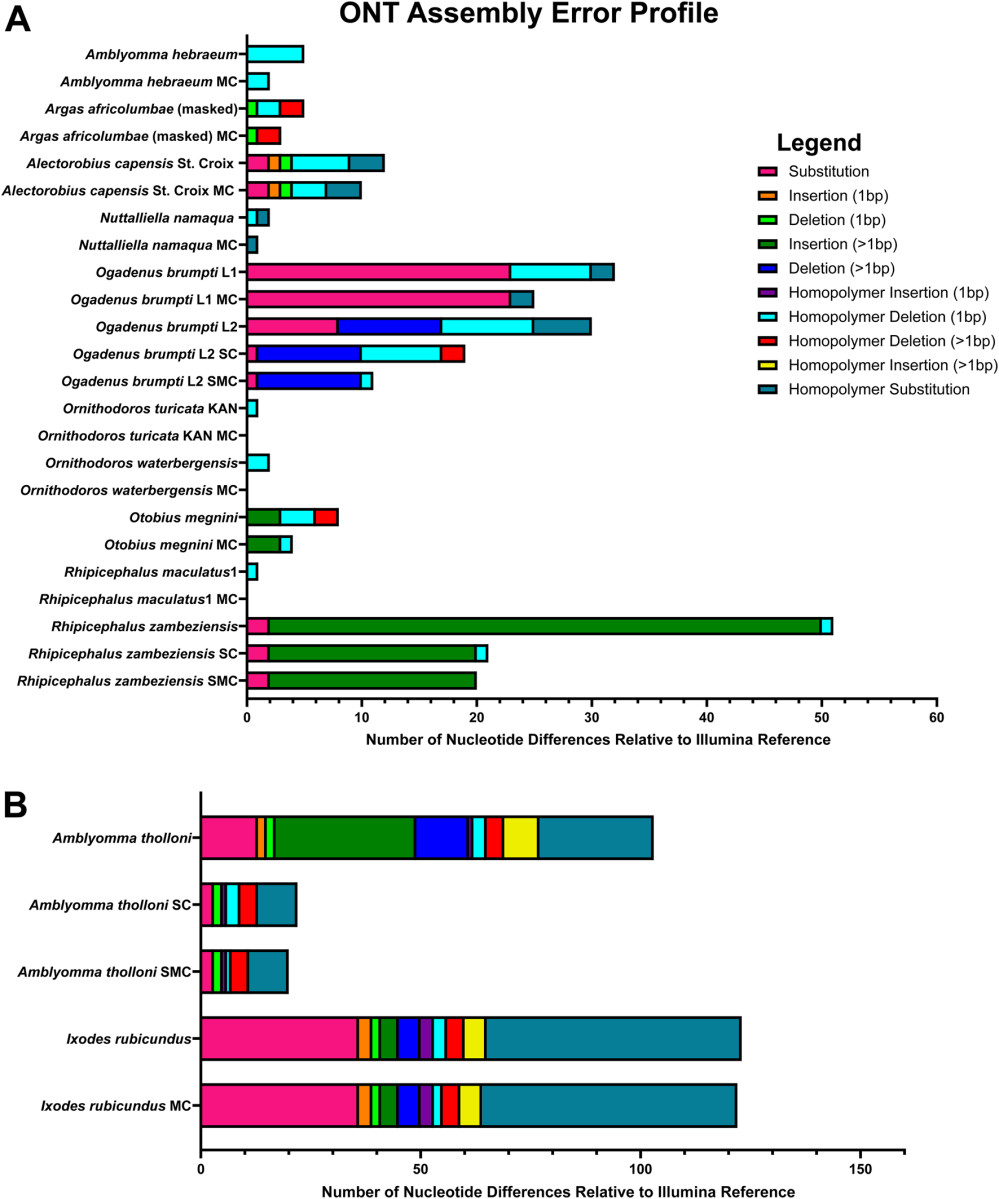
ONT assembly error profile. We characterized the nucleotide differences between the ONT assemblies and the Illumina references to determine an error profile. The error profile was plotted in two different groups, those assemblies with < 100 bp differences (**A**) and those with > 100 bp differences (**B**). The number of nucleotide differences is classified for each assembly and after any error correction steps taken. Homopolymers are defined as any span of the same nucleotide > 2. *SC* Sanger correction, *MC* manual correction, *SMC* Sanger correction followed by manual correction.

We also characterized nucleotide differences between the ONT and Illumina assemblies. The differences that occurred for each ONT assembly relative to the Illumina reference were inspected and we developed an error profile for each benchmark sample (Fig. 3). The error profile varied widely between samples. The NucDiff assemblies of *A. tholloni* 1, *Ogadenus brumpti* L2, and *R. zambeziensis* had clusters of nucleotide differences in repeat regions, large deletions, or areas of generally poor alignment. This suggested that the Illumina reference for these samples may have contained errors. We determined which assembly was correct by amplifying and performing Sanger sequencing on areas that had clusters of nucleotide differences between the ONT and Illumina assemblies on the *A. tholloni* 1, *O. brumpti* L2, and *R. zambeziensis* samples. In all cases but one, the Sanger sequencing data supported the ONT assembly (Supplementary File 1). The one region that was not supported by Sanger sequencing was a nine-base deletion in the *cox1* gene of *O. brumpti* L2. This deletion was not found in the *O. brumpti* L1 assembly. Overall, the mean and median sequence agreement between the ONT assemblies and Illumina reference assemblies (including manual corrections to the ONT assemblies and Sanger corrections to Illumina assemblies) was 99.89% and 99.98%, respectively.

### Mitophylogenomic analysis

**Figure 4:**
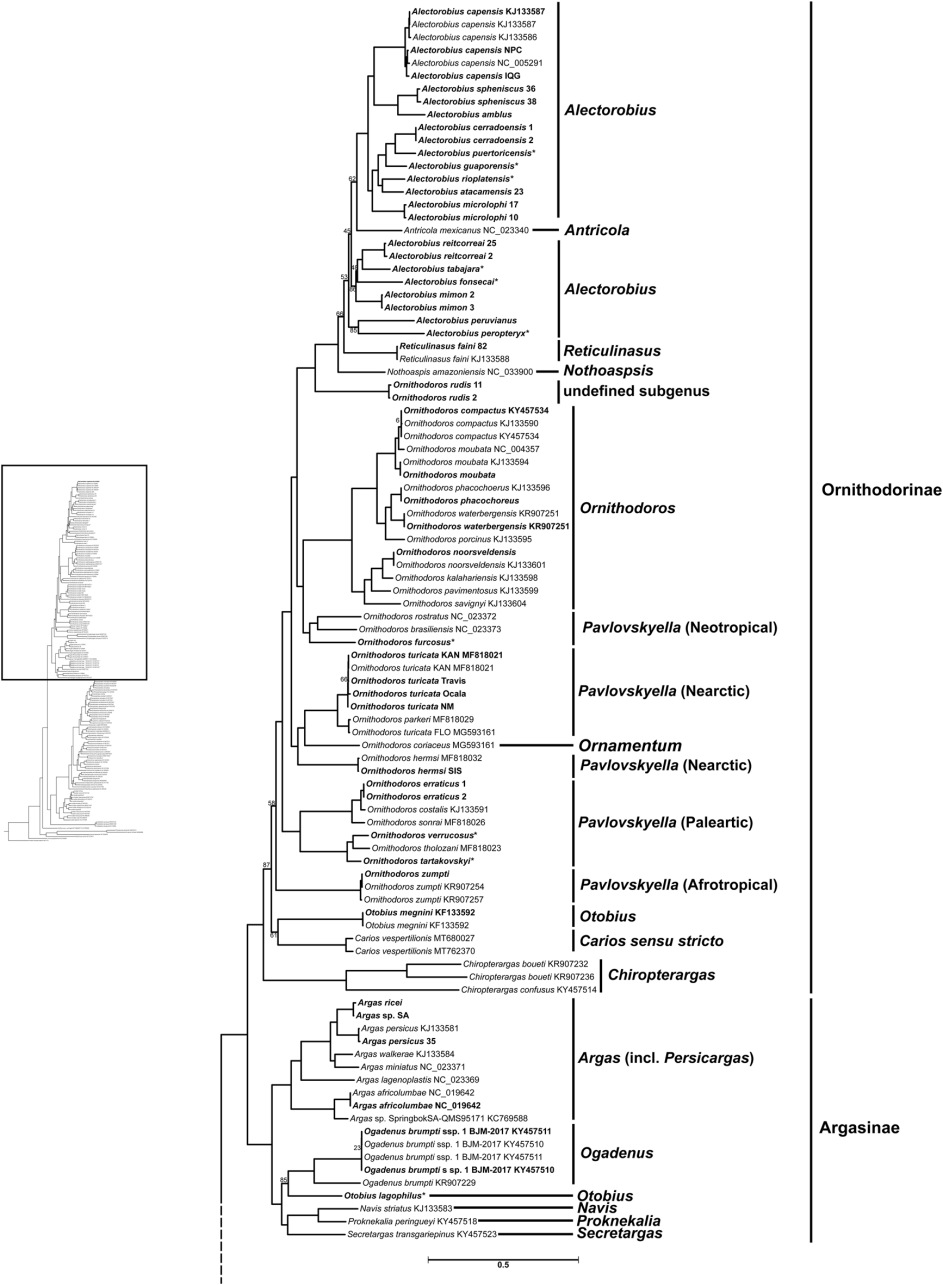
Argasidae phylogeny. The Argasidae portion of the maximum likelihood inferred phylogeny using the 10AA scheme from Kelava et al*.*^12^ for the Argasidae, Nuttalliellidae, and Ixodidae is shown here. Ultra-fast bootstrap support is reported on branches as the percentage of 100,000 replicates, those with < 90% support are shown. Tip labels that contain an asterisk (*) indicate that there were multiple samples of the same species, from the same locality, whose sequence alignment was 100% the same. GenBank accession numbers are located at the end of tip labels. Tip labels that are bolded indicate samples that were sequenced in this study and those that are bolded with GenBank accession numbers were a part of the benchmarking cohort. The subgenus that the tips correspond with are indicated to the right. The subfamilies are indicated to the right of the subgenus. The scale bar indicates number of substitutions per site. We include a phylogeny to the left to orient the reader to which part of the total tick phylogeny they are viewing. The dotted branch indicates where this portion of the phylogeny meets the rest of the phylogeny.

**Figure 5:**
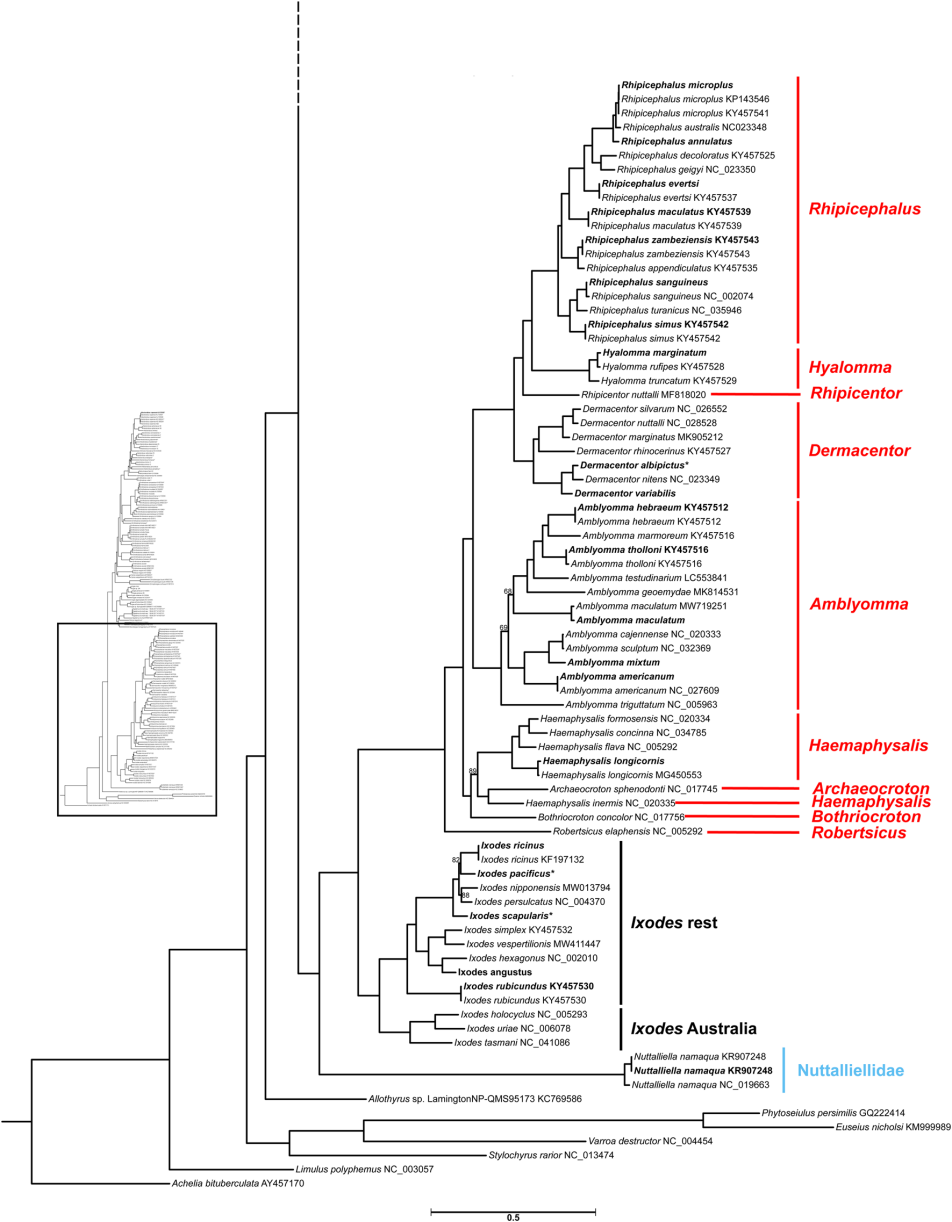
Ixodidae and Nuttalliellidae phylogeny. The Ixodidae and Nuttalliellidae portion of the maximum likelihood inferred phylogeny using the 10AA scheme from Kelava et al*.*^12^ for the Argasidae, Nuttalliellidae, and Ixodidae is shown here including the root taxa. Ultra-fast bootstrap support is reported on the branches as the percentage of 100,000 replicates, those with < 90% support are shown. Tip labels that contain an asterisk (*) indicate that there were two samples of the same species, from the same locality, whose sequence alignment was 100% the same. GenBank accession numbers are located at the end of tip labels. Tip labels that are bolded indicate samples that were sequenced in this study and those that are bolded with GenBank accession numbers were a part of the benchmarking cohort. The Metastriates genera are indicated in red, the Prostriates genus subgroups are indicated in black, and the Nuttalliellidae family is indicated in blue. The scale bar indicates number of substitutions per site. The phylogeny to the left indicates the portion of the complete tick mitogenome phylogeny being viewed. The dotted branch indicates where this portion of the phylogeny meets the rest of the phylogeny.

We phylogenetically analyzed the generated mitogenomes in the context of all major groups of the three tick families. Using the 10AA scheme from Kelava et al*.*^12^, we inferred a maximum likelihood tree from our dataset that was largely congruent with mitophylogenomic analyses previously reported^10–12^ (Figs. 4, 5). Our analysis found that many of the new mitogenome sequences from South America clustered with the *Alectorobius* genus further increasing the monophyly of a Neotropical clade of species with similar morphology and developmental traits. *Ornithodoros rudis* formed a well-supported distinct clade of its own (100% bootstrap support) and could not be placed in a specific subgenus based on the 10AA scheme used. In Nearctic *Pavlovskyella*, we observed that a recently established *Ornithodoros turicata* colony that originated from ticks collected from Ocala, Florida (*O. turicata* Ocala) clustered with *O. turicata* and not *Ornithodoros parkeri*^26,27^*.* This was surprising because previous *O. turicata* specimens from Florida (*O. turicata* FLO) phylogenetically clustered with *O. parkeri*^10^. We further investigated this finding by performing whole-mitogenome alignment and determined the pairwise sequence identities against representatives of *O. turicata* and *O. parkeri* (Supplementary Table S6)*.* We found that *O. turicata* Ocala had 98.9% sequence identity to other *O. turicata* specimens while having only 86% sequence identity to *O. parkeri*. The species delimitation point reported by Mans et al*.* for whole-mitogenome pairwise sequence analysis was > 95%^10^. This indicates that the *O. turicata* Ocala colony is indeed *O. turicata*. Mans et al*.* also reported pairwise sequence identities of *O. turicata* FLO with *O. turicata* and *O. parkeri* specimens as well^10^, which indicated that *O. turicata* FLO is likely *O. parkeri*. Furthermore, we observed the placement of *Otobius lagophilus* in the Argasinae subfamily and not in the Ornithodorinae subfamily with *Otobius megnini*. These data indicate that the *Otobius* genus is likely paraphyletic.

## Discussion

This work described a strategy for full-length amplification, sequencing, and assembly of mitogenomes from individual tick specimens using ONT MinION technology. Our goals were achieved for this study in terms of cost per sample and accuracy. The assemblies generated had a cost per sample of ~ $10 USD (Supplementary Table 7). This is similar to 2 × coverage of an 800 bp to 1 kb amplicon sequenced by Sanger methods. Further, the ONT assemblies benchmarked well compared to those generated by a previously published Illumina genome skimming strategy^10^.

We achieved concordant mitogenome assemblies compared to published Illumina assemblies of the same sample^10^. By leveraging the R10.3 flow cell and the super-accuracy model in Guppy we generated assemblies that had a median sequence concordance of 99.98% compared to assemblies generated by Illumina genome skimming of the same sample. However, we observed that in 4 of 5 regions chosen for Sanger sequencing, the ONT assembly had the correct sequence versus the Illumina reference suggesting the former strategy may be advantageous. These errors in the Illumina references made it difficult to establish a ground truth for benchmarking and thus we cannot guarantee the accuracy of the benchmarking in this study.

We characterized the most common errors causing frameshifts in protein coding regions in the ONT assemblies. These errors were almost exclusively single-base deletions in long A/T homopolymer regions (> 8 bp). Homopolymer errors are known issues with ONT sequencing^28^, though the R10.3 pore type is supposed to offer enhanced accuracy compared to the R9.4.1 flow cell^29,30^. Manual annotation was able to correct these frameshift-causing homopolymer errors.

We also observed that both the P1 and P2 amplicons need to be sequenced for a complete mitogenome. This was highlighted by the *A. africolumbae* and *I. rubicundus* assemblies. Depending on the question at hand however, use of one primer set may be sufficient if the gene feature containing the primer site can be excluded from analysis. Despite what errors may have occurred in our assemblies, the data were phylogenetically consistent with previous work using orthologous sequencing modalities demonstrating this strategy’s utility.

Results from this mitophylogenomic analysis were consistent with previous reports^10–12^, and increased the evidence for the monophyly of a large Neotropical group of species sharing morphological and developmental characters consistent with *Alectorobius* sensu Clifford et al*.*^31^. Most members of *Alectorobius* have a Neotropical distribution and parasitize a variety of organisms including mammals, reptiles, bats, and birds^32^. The addition of new mitogenome data from 15 Neotropical species (described as *Ornithodoros* spp.) increased the species within *Alectorobius* phylogenetically, and highlights that the genera *Antricola*, *Nothoaspis*, and *Reticulinasus* could also be linked to this genus. Indeed, available biological and morphological data for those four taxa show that they have slow-feeding larvae and cheeks present in adults^32^. However, bootstrap support was weak (52–68% bootstrap support) for deeper relationships within the *Alectorobius*. Increased taxonomic sampling of *Alectorobius* spp. and especially *Alectorobius talaje*, the type species of the genus, will likely help to resolve these relationships.

Our analysis placed *O. rudis* sharing a common ancestor with what has previously been referred to as *Carios *sensu lato^33^. Morphologically larvae of *O. rudis* resemble and behave like *Pavlovskyella* since they feed in minutes and the first nymphal instar needs a blood meal to molt^34^. However, adults exhibit *Alectorobius* traits such as cheeks, but lack a dorsoventral groove or tarsal humps^32^. These ambiguities have precluded *O. rudis* from being confidently placed in a subgenus^32,35^. Our molecular findings here further support the unique phenotypic nature of *O. rudis*, placing it in a unique lineage that would likely represent a separate genus.

Our mitophylogenomic analysis also revealed that the *O. turicata* specimen from Ocala, Florida^26,27^ was more closely related to *O. turicata* than *O. parkeri.* This contrasts with prior work with *O. turicata* samples from Florida (*O. turicata* FLO) previously reported by Mans et al.^10^. Our pairwise sequence identity data further supported the placement of *O. turicata* Ocala in the *O. turicata* species. Our result indicates that within Florida there may exist sympatric populations of *O. turicata* and *O. parkeri* or a hybrid of the two. Future population genetics studies on the *Ornithodoros* species of Florida are needed to investigate the circulation of different species in the state.

This work generated the first *O. lagophilus* mitogenome and determined its phylogenetic relationship with other tick species. Interestingly, *O. megnini* (the only other species described in the *Otobius* genus) has been an unstable taxon in other phylogenetic analyses of ticks^10–12^. We hypothesized that by including *O. lagophilus* in our analysis we would better resolve the placement of the *Otobius* genus. However, *O. lagophilus* was placed with the Argasinae to the exclusion of *O. megnini*. These findings, based on mitochondrial loci, suggest that the *Otobius* genus is paraphyletic. Since *O. megnini* is the type for the genus *Otobius*, it would place *O. lagophilus* in its own genus. Future work examining nuclear loci would be informative for helping to discern the genetic relationship between *O. lagophilus* and *O. megnini*.

Limitations of this work involved the variability in primer success, the establishment of a ground truth for the mitogenome sequence, and the error rate of the ONT sequencing technology. The possibility that some of the reference samples may have erroneous identities can also not be excluded. While we amplified whole mitogenomes from all three tick families, 14 different genera, and 68 different species, the primers did not work universally. This may be due to several factors; however, we believe two likely contributed greatly. The first was sample quality and the second was primer site mismatch. This strategy uses inverted primers and thus requires an intact circular mitogenome. Moreover, because of the length of the PCR, sample purity is also a concern. The genomic DNA samples used in this study came from different sources, extraction methods, and varied in terms of age and sample quality (i.e. NanoDrop ratios). Tick DNA was extracted from fresh specimens, specimens stored in ethanol, or specimens frozen dry at − 80 °C. In some instances, sub-optimal DNA quality may have compromised the PCR, failing to yield an amplicon. However, we amplified DNA samples that were approximately 5 years old, so this strategy is feasible on older samples^10^. We would caution the use of genomic DNA extracted by bead beating methods since this could possibly decrease the amount of circular mitogenome in the sample. Fresh specimens extracted by column- or magnetic bead-based strategies could be best; however, we did not empirically determine the best specimen storage or extraction method(s) for this protocol.

Primer site mismatch is also a likely reason for PCR failure. While we developed degenerate primers from a wide taxonomic range of tick mitogenomes, it is unlikely that universal primer sites would exist. We envision that the primers we developed here can be used as a first step when little a priori knowledge of the specimen is available, and that genus or species-specific primers could be designed as needed for enhanced success. We demonstrated the utility of ad hoc species-specific primers for generating amplicons where the generic primer sets initially failed.

ONT sequencing is known as being error prone; however, this was only problematic in homopolymeric regions. We consider the ONT error rate a minor limitation as ONT is a developing technology that is rapidly approaching the accuracy of Illumina sequencing^29,36^. Indeed, we showed examples where with the use of Guppy (v5.0.7) sup basecalling and the R10.3 pore, ONT sequencing was capable of being more accurate than Illumina. It is likely that the recent improvements of ONT sequencing (R10.4.1 flow cell chemistry, coupled with kit14 Q20 + library preparations and the latest basecalling models) will address many of the errors seen in our data. Despite the limitations indicated, we were still able to generate high-quality, mitogenomes from a wide variety of tick taxa and generated mitogenome sequence data for 26 tick species not currently represented on GenBank.

Ticks are important organisms that contribute to the global disease burden of humans and other animals alike. A better understanding of their distributions, taxonomy, and genetic diversity will help to inform countermeasure strategies. Mitogenome analysis is a space that can contribute to such an understanding. The work described here is a low cost, high accuracy strategy for amplifying and sequencing the entire mitogenome from an individual tick specimen. We demonstrated the utility to scale, sequencing 72 samples in a single library prep and generated 85 individual mitogenomes from 57 different tick species. Twenty-six of these species were previously without a complete mitogenome on GenBank. This method will be important to furthering our understanding of tick evolution, dispersal, population genetics, and could likely be applied to other metazoans.

## Methods

### Tick classification schemes

The classification scheme of Guglielmone et al*.*^2^, with updates on hard tick genus classifications by Barker and Burger^37^ for *Archaeocroton* and *Robertsicus* was used. For the soft ticks, the classification scheme of Mans et al.^10,11^ was used.

### Tick samples

#### Tick specimen sourcing

Tick specimens were provided from several sources identified in Supplementary Table S8. All samples were morphologically and, in some cases, molecularly identified by the providing lab. The following reagents were provided by Centers for Disease Control and Prevention for distribution by BEI Resources, NIAID, NIH: *Ixodes ricinus* Adult (Live), NR-42511; Adult *Dermacentor variabilis*, NR-42513; *Ixodes scapularis* Adult (Live), NR-42510; Adult *Rhipicephalus sanguineus*, NR-42512; Adult *Haemaphysalis longicornis*, NR-51846; *Ornithodoros tartakovskyi* Adult Female Live, NR-48929; *Ixodes pacificus* Adult (Live), NR-44385. The following reagents were provided by the Centre de coopération Internationale en Recherche Agronomique pour le Développement (CIRAD) for distribution by infraVec2: *Hyalomma marginatum* preserved or extracts (ref# V.11.1.P.FR.7); *Ornithodoros moubata* preserved or extracts (ref# V.9.2.P.FR.7); *Ornithodoros erraticus* preserved or extracts (ref# V.9.1.P.FR.7.26).

#### Genomic DNA isolation

For samples from SML, MBL, PDT, JEL, SF, RJE, AAPL, KL, TS, IM, BEI, and IV2 (see Supplementary Table S8), genomic DNA from individual tick specimens was isolated using Qiagen’s DNeasy Blood and Tissue kit following manufacturer’s directions with the following modifications: tick samples were macerated with a sterile plastic pestle in the ATL buffer with proteinase K and incubated overnight at 56 °C in a rotating incubator. The optional 56 °C step for 10 min after the addition of AL buffer was performed for all samples, and DNA was eluted in 35 µL of prewarmed (56 °C) EB buffer (Qiagen). The *Ornithodoros tabajara* samples were small larvae and were processed using a modified squish buffer protocol^38^ outlined in Supplementary File 2. Genomic DNA samples provided by SML* and MBL*^39^, RJE^40^, and AB, AL, DGK, EK, MS, RP, TGS, BJM^10^ were extracted as previously described.

### Full-length amplification of tick mitogenomes

Tick mitogenomes are circular but exist in two different organizations^20^; because of this, we designed two different series of inverted PCR primers. Group A consisted of the argasid, nuttallielid, and prostriate ticks. Group B consisted of metastriate ticks. Degenerate primers were manually designed by inspection of whole mitogenome alignments within each group using CLUSTAL Omega^41^ (see Supplementary Table S9 for a list of mitogenomes used to identify the primer sites). Primer sites were chosen based on conservation across a wide variety of taxa while also minimizing the number of degenerate sites within each primer set (P1 and P2). In instances where our general primers failed but a mitogenome for that species was available, we designed species-specific primers to minimize degeneracy and mismatches (species-specific, ssP1 and ssP2). All primers used in this study as well as their annealing temperatures are shown in Supplementary Table S1. Because the samples were provided in varying volumes and concentrations the inputs for PCR ranged from 6 to 100 ng of total genomic DNA. The PCRs were carried out with either a C1000 (BioRad) or MiniAmp Plus (Applied Biosystems) thermal cycler using Platinum SuperFi 2 × master mix (Invitrogen) with modified cycling conditions: (95 °C, 1 min) 1 cycle, (95 °C, 10 s; see Supplementary Table S1 for annealing temperatures, 15 s; 68 °C, 15 min) 30 cycles, (68 °C, 7 min 30 s; 12 °C, hold). Negative controls were included for each primer set. All PCR samples were analyzed via gel electrophoresis using 0.8% agarose: tris–acetate-EDTA (TAE) gels with 1 × GelRed (Biotium) at 120 V for 1 h and imaged on a ChemiDoc MP (BioRad). A definite band at ~ 15 kb was required for the sample to be considered successful. In cases where strong bands were below 15 kb, these samples were considered failed reactions. All successful reactions and negative controls were quantified with a Qubit 4 using the 1 × dsDNA HS kit (Invitrogen) per manufacturer’s instructions with one microliter of sample. The samples were then stored at 4 °C until library preparation.

### ONT library preparation and sequencing

Samples and negative controls were prepared with either the SQK-RBK004 or SQK-RBK110.96 library preparation kits following manufacturer’s instructions with the following modifications. For the SQK-RBK004 library, the primer set 1 (P1) and primer set 2 (P2) amplicons were pooled equimass to 260 ng per sample and purified using Mag-Bind Total Pure NGS beads (Omega Biotek) in a ratio of 1:1 (v/v) beads to sample volume and followed manufacturer’s instructions for purification in a 96 well PCR plate (Applied Biosystems), eluting with 10 µL of water. With samples that only had a P1 or P2 amplicon, the entire 260 ng consisted of the one amplicon. For cases where there was less than 130 ng of one amplicon, the remainder was made up of the other amplicon so all samples consisted of a final 260 ng of pooled amplicon DNA. For negative controls, the entire sample of the P1 and P2 PCRs were pooled and purified. For the barcoding reaction, 7.5 µL of the P1 and P2 pooled amplicon elution was used for the barcoding reaction of each sample. One microliter from the elution was quantified with a Qubit 4 using the 1 × dsDNA HS kit (Invitrogen), this quantification was used to normalize the amount of each barcode that was pooled together. All barcodes were pooled equimass such that 120 ng from each barcoding reaction was pooled. The pooled samples were purified with Mag-Bind Total Pure NGS beads (Omega Biotek) beads in a 1:1 (v/v) ratio. The beads were washed twice with long fragment buffer (LFB) and then once with 70% ethanol. The rest of the SQK-RBK004 protocol was followed per manufacturer’s instructions. In the SQK-RBK110.96 library preparations, 200 ng of P1 and P2, or ssP1 and ssP2 PCRs were pooled equimass to 200 ng and purified, eluted, and quantified as before for the SQK-RBK004 library prep. The entire barcoding reaction was pooled for each sample and the remainder of the protocol was followed per manufacturer’s instructions. Both the SQK-RBK004 and SQK-RBK004 library preparations were sequenced with an R10.3 pore MinION flow cell on an Mk1B sequencer.

### Sequence analysis

#### Example commands and scripts

Example commands and custom or modified scripts are found in Supplementary File 3.

#### Sequence data preparation

The ONT FAST5 raw sequencing files were basecalled separately for each library using ONT’s Guppy v5.0.7 and the “super-accuracy” basecalling model generating FASTQ files of raw, basecalled data. The raw, basecalled data were demultiplexed using guppy_barcoder with the “-detect_mid_strand_barcodes” option. After demultiplexing, the data were initially processed using NanoFilt (v2.7.0)^42^ to remove all reads less than 500 bp and greater than 17.5 kb, as well as, reads with quality scores less than 10. Summary statistics for each sample’s ONT data were generated using NanoStat (v1.2.0) (Supplementary Table S4)^42^.

#### Assembly and polishing

The filtered sequencing data for each sample was assembled using Flye. Assemblies were first attempted using Flye (v2.8.3-b1695) with the -meta and -t 35 option, if this failed the assembly was repeated with threads (-t) set to 8^43,44^. Assemblies that still failed were assembled with Flye (v2.9-b1774) with the -nano-raw and -meta options. Those assemblies assembled with Flye v2.9 are indicated in Supplementary Table S4. The largest contig of each assembly was extracted and subjected to a modified version of the apc.pl script^45^ (apc_mod.pl) to remove overlapping circular sequence. The output of the apc_mod.pl script was polished using the initial assembly’s respective version of Flye with three iterations (-i 3) followed by Medaka (v1.4.3) polishing once. The assembly was then reoriented to the methionine tRNA in the plus-sense using BLASTn (v2.12.0)^46^ with a close relative’s methionine tRNA sequence.

#### Automated and manual annotation

Annotation was done using MitoZ (v2.3)^23^ using the “annotate” module. The GENBANK files generated by MitoZ were visualized and manually corrected in Geneious Prime (v2021.2; Biomatters). Manual annotation correction was done to reduce gene overlaps between gene features. The tRNA annotations were confirmed, or added if a tRNA was missing, using the online ARWEN server with only the -mtx option selected^24^. Frameshift mutations resulting in premature stop codons in protein coding genes were also manually corrected. Correction was almost always achieved by the addition of a single base to the homopolymer upstream of the premature stop codon. In some cases, larger deletions were corrected to account for size differences between mitogenome assemblies of specimens from the same colony (e.g. *Ornithodoros erraticus* 1) when supported by read alignment data. In the case of missing genes (e.g. *atp8* in the *H. longicornis* sample), these were annotated using BLASTn of a close-relative’s sequence of said gene. All manual modifications of assembly sequences are outlined in Supplementary File 4. Mean depth of coverage reported in Supplementary Table S4 was determined using minimap2 (v2.22-r1101)^47^ and mosdepth^48^ performed on the corrected assemblies using respective read dataset used for the assembly.

#### Benchmarking

Benchmarking was performed with NucDiff (v2.0.3)^25^ using the GenBank assembly from Mans et al*.*^10^ as the reference and the ONT assembly of the same sample as the query. The insertion, deletion, and substitution events were reported as well as the overall number of nucleotide differences. Sequence accuracy was calculated by subtracting the quotient of the total nucleotide differences divided by the reference assembly size from one then multiplying by 100%. Masking of the amplicon ends for *A. africolumbae* JM2 was done by mapping the ONT reads to the Illumina assembly from GenBank using minimap2 and Geneious Prime to visualize said alignment. The nucleotide differences that fell within the region of the Illumina assembly that had no ONT read coverage were not considered in the accuracy calculation during masking.

#### Mitophylogenomic analysis

Manually corrected mitogenome assemblies were exported from Geneious Prime as an ASN file using the GenBank Submission tool. The ASN file was converted to a multi-GENBANK file using the asn2gb tool from NCBI. The multi-GENBANK file was split into individual GENBANK files and the amino acid sequences of the protein coding genes were extracted using splitgbk.py and gbk_to_faa.py respectively. Mitogenome sequences obtained from NCBI were downloaded as GENBANK files and the amino acid sequences were extracted with gbk_to_faa.py. All amino acid sequences were combined and the sequences for each protein were extracted using grep. Protein sequences were aligned using MAFFT (v7.486) with G-INS-i option^49^. The alignments were trimmed of variable alignment sites and sites containing greater than 50% gaps using Gblocks (v0.91b)^50^.

Phylogenies were inferred using IQ-TREE2 (v2.1.2) with an edge-linked partition scheme using alignments from the 10AA dataset (ATP6, COX1, COX2, COX3, CYTB, NAD1, NAD2, NAD3, NAD4, NAD5)^12,51,52^. The protein sequence alignments used for tree inference are available in Supplementary Files 5–14. We used 100,000 ultrafast bootstrap replicates, set the root to *Achelia bituberculata* with -o option, and automated model selection with the -m MFP option^53,54^. The optimum substitution model determined for each protein alignment was as follows: ATP6, mtMet + F + R6; COX1, mtART + R5; COX2, mtMet + R6; COX3, mtInv + R6; CYTB, mtMet + R6; NAD1, mtZOA + F + R7; NAD2, mtMet + F + G4 ; NAD3, mtART + I + G4; NAD4, mtInv + R6; NAD5, mtInv + F + R7. The Newick tree file generated from IQ-TREE2 is available in Supplementary File 15. The phylogeny was visualized in iTOL (v5)^55^ and annotated with Inkscape (v1.2)^56^.

#### Whole-mitogenome pairwise sequence identity calculation

Whole-mitogenome pairwise sequence identities were calculated in Geneious Prime. Using the MAFFT tool in Geneious Prime with default settings (Algorithm: Auto, Scoring matrix: 200PAM/k = 2, Gap open penalty: 1.53, Offset value = 0.123), we performed pairwise alignments of the assemblies shown in Supplementary Table S6 and reported the pairwise identity calculated by Geneious Prime.

### Sanger verification of nucleotide differences

We identified regions that contained multiple nucleotide differences between the ONT assemblies and the Illumina reference by first performing BLASTn analysis and then confirming these regions by mapping the ONT reads against the Illumina reference using minimap2. To verify which assembly was correct we performed PCR and Sanger sequencing. The regions were amplified using 40 ng of genomic DNA with Platinum SuperFi 2 × master mix (Invitrogen) with cycling conditions: (98 °C, 30 s) 1cycle, (98 °C, 10 s; see Supplementary Table S1 for annealing temperatures, 15 s; 72 °C, 30 s) 35cycles, (72 °C, 5 min; 12 °C, hold). Negative controls were included for each primer set. The PCRs were analyzed via gel electrophoresis using 0.8% agarose: tris-acetate-EDTA (TAE) gels with 1 × GelRed (Biotium) at 120 V for 1 h and analyzed on a ChemiDoc MP (BioRad). The PCR amplicons were purified with Mag-Bind Total Pure NGS beads (Omega Biotek) beads following manufacturer’s instructions in a 1:1 (v/v) ratio with the sample. Purified amplicon was sent to Genewiz (South Plainfield, NJ, USA) for Sanger sequencing in both directions using the same forward and reverse primers used in the PCR. Sanger chromatograms were analyzed using FinchTV (v1.4.0, Geospiza, Inc.; http://www.geospiza.com) and the sequence data were analyzed in Serial Cloner (v2.6.1, Serial Basics, http://serialbasics.free.fr/Softwares.html). The consensus sequence of the forward and reverse Sanger data was aligned against the Illumina reference and ONT assembly to determine which sequence was correct (Supplementary File 1).

## Supplementary Information

Supplementary Information 1.

Supplementary Information 2.

Supplementary Information 3.

Supplementary Information 4.

Supplementary Information 5.

Supplementary Information 6.

Supplementary Information 7.

Supplementary Information 8.

Supplementary Information 9.

Supplementary Information 10.

Supplementary Information 11.

Supplementary Information 12.

Supplementary Information 13.

Supplementary Information 14.

Supplementary Information 15.

Supplementary Information 16.

## Data Availability

All sequencing data generated in this study is associated with the NCBI BioProject PRJNA837010. The read data is available on GenBank’s sequence read archive (SRA accessions SRR19325806-SRR19325892) through the associated BioSample (BioSample accessions SAMN28408697-SAMN28408766, SAMN28512635-SAMN28512649, SAMN28534441, and SAMN28534442) for each specimen sequenced. The BioSample accession number for each specific specimen is found in Supplementary Table S4.
