# Supplementary material for: Amplification and sequencing of entire tick mitochondrial genomes for a phylogenomic analysis
Source: Sci Rep. 2022 Nov 11;12:19310. doi: 10.1038/s41598-022-23393-5 (PMC9652274; doi:10.1038/s41598-022-23393-5)
Supplement: Supplementary file 2 — Supplementary Information 2. [file 41598_2022_23393_MOESM2_ESM.pdf]

Squish Protocol for gDNA Isolation from Small Specimens

Materials

- 1.5ml Microtube
- 0.2mL PCR tube
- P200 micropipettor
- P20 micropipettor
- Hula tube mixer
- 70% EtOH
- Squish Buffer (make fresh)
  - 10mM Tris-HCl pH 8.0
  - 1mM EDTA
  - 25mM NaCl
  - 200ug/mL Proteinase K

- Omega BioTek Mag-Bind TotalPure NGS magnetic beads (OmegaBiotek M1378-00)
- 1.5mL tube magnet for bead purification
- Incubator/thermal cycler for 37°C incubation
- 56°C preheated buffer EB (Qiagen)
- Centrifuge

Procedure

1. Place specimen in a 0.2mL PCR tube
2. Crush specimen thoroughly with a p200 with a 200uL pipette tip containing 50uL of squish buffer (use appropriate amount of volume depending on specimen, very small tick larva can be crushed and extracted in 20uL of sample with a p20, p200/50uL would be the upper end of the spectrum for something like an adult mosquito or larger tick nymph).
3. After crushing the specimen expel the squish buffer that remains in the pipette tip
4. Incubate the tube at 37°C for 30min in thermal cycler/incubator
5. Transfer digestion to 1.5mL microtube and add 0.8x vol of Omega BioTek Mag-Bind TotalPure NGS magnetic beads
6. Mix digestion and beads together gently but thoroughly by finger vortexing/flicking
7. Incubate digestion and beads at room temp for 10mins on a Hula mixer
8. Place digestion and beads on magnet for 2mins
9. Remove supernatant and wash beads with 200uL of fresh 70% EtOH
10. Repeat step 8-9
11. Remove EtOH and pulse spin tube to pellet beads
12. Place tube on magnet for 2mins and remove remaining EtOH
13. Let tube dry for 2mins
14. Add 20uL preheated (56°C) buffer EB
15. Incubate on the bench for 10mins
16. Place tube on magnet for 2mins
17. Remove elution to new tube

Adapted from http://francois.schweisguth.free.fr/protocols/Single_fly_DNA_prep.pdf
